# Pb(ΙΙ), Cd(ΙΙ), and Mn(ΙΙ) adsorption onto pruning-derived biochar: physicochemical characterization, modeling and application in real landfill leachate

**DOI:** 10.1038/s41598-024-54028-6

**Published:** 2024-02-10

**Authors:** Maryam Rabiee Abyaneh, Gholamreza Nabi Bidhendi, Ali Daryabeigi Zand

**Affiliations:** 1 Department of Environmental Engineering, University of Tehran, Kish International Campus, Kish, Iran; 2 Faculty of Environment, University of Tehran, Tehran, Iran

**Keywords:** Biochar, Wood waste, Adsorption, Pyrolysis temperature, Landfill leachate, Heavy metals, Environmental sciences, Chemistry

## Abstract

The aim of this study was to systemically evaluate how different pyrolysis temperatures (400, 550, and 700 °C) and particle sizes (1–2 mm and 63–75 µm) were influenced biochar evolution, made from urban pruning waste, during pyrolysis process and to establish their relationships with biochar potential for removal of lead (Pb), cadmium (Cd), and manganese (Mn) from real municipal solid waste landfill leachate. The effects of pH (2–7), contact time (30–300 min) and adsorbent dosage (0.1–5 g L^−1^) on heavy metals removal were also examined. The results showed that physicochemical properties of biochar were greatly influenced by pyrolysis temperature. Particle size, however, showed little influence on biochar characteristics (*p* > 0.05). The yield, volatile matter, hydrogen and oxygen contents, and surface functional groups decreased consistently with increasing pyrolysis temperature. An increase in the pH, electrical conductivity, ash, fixed carbon, and specific surface area values was also found. In biochar samples formed at high temperatures (i.e., 550 and 700 °C), Fourier transform infrared spectroscopy-FTIR studies confirmed the increase in aromaticity. Field emission scanning electron microscopy-FESEM images showed differences in the microporous structure and lower size pores at higher temperatures. Biochar pyrolyzed at 700 °C with a particle size of 63–75 µm (i.e., Lv700-63) showed the highest removal efficiency performance. Pb and Cd ions were completely removed (100%) by 0.2 g L^−1^ Lv700-63 at 7.0 pH and contact times of 120 and 90 min, respectively. The maximum percentage removal of Mn was 86.20% at optimum conditions of 0.2 g L^−1^ Lv700-63 dosage, 7.0 pH, and 180 min contact time. The findings suggests that the surface complexation, π-electron coordination, and cation exchange were the dominant mechanisms for the Pb, Cd, and Mn removal onto Lv700-63.

## Introduction

Population growth and urbanization have led to increasing municipal solid waste (MSW) production over the past decades^1^. Despite progresses made in different disposal and recycling techniques, burying waste in the ground is still a common method worldwide^2^. Economic benefits of waste burial in landfills make this method an attractive ultimate disposal option for MSW management, particularly in developing countries, where implementing desirable waste disposal options such as recycling and waste to energy require extensive planning and large investment to provide infrastructures^3^.

Leachate production is a major problem associated with burying MSW in the ground. Different organic, inorganic, and xenobiotic compounds may exist in the MSW landfill leachates^4^. So, the removal of contaminants has become an urgent problem in leachate treatment. As the landfill aging, the biological decomposition of deposited waste will reduce and leachate will become more stable due to the present of bio-refractory compounds^5^. Accordingly, the biodegradable factor of leachate plummets as the age of landfill increases. Thus, the old landfill leachate composition is more complicated than the young landfill leachate, as it is impossible to effectively treated with biological processes^6^.

There are different techniques for the removal of diverse pollutants from old landfill leachate, such as chemical precipitation^7^, adsorption^8^, oxidation^9^, evaporation^10^, reverse osmosis^11^, and ion exchange^12^. Adsorption is one of the most successful methods used to remove a variety of compounds from wastewaters^13^. Adsorption using commercial activated carbon (CAC) have been widely studied as efficient adsorbent for the removal of HMs^14^. CAC is a form of carbon processed to have small, low-volume pores that increase the surface area available for adsorption or various chemicals^15^. CAC illustrates significant adsorption ability in gas and liquid phases due to its high micropore volume, large specific surface area (SSA), favorable pore size distribution, thermal stability and capability for rapid adsorption and low acid/base reactivity. Hence, CAC has a great capability to remove essential amounts of inorganic compounds such as HM ions from the landfill leachate^16,17^. Despite the advantages of adsorption method, some disadvantages including high preparation and recovery costs and the need for specialists, limited its use in many cases^18^. Thus, extensive research has been performed to find a low-cost alternative to CAC with high adsorption capacity so as to not only decrease the adsorbent dosage and the associated required costs but also increase the adsorption efficiency to the extent possible^19^.

Biochar is a carbon-rich material produced from the thermochemical decomposition of biomass under limited or no oxygen conditions^20^. Biochar is a substance with high porosity and SSA that has many functional groups and is very effective in the adsorption of HMs, particularly in aqueous solutions. Therefore, many researchers have recently become interested in using biochar as a low-cost alternative to CAC offering a new strategy for water and wastewater treatment^21^. Agricultural and industrial wastes such as coconut shell^22^, *Sedum alfredii*^23^, olive stone^24^, sewage sludge^25^, and red mud^26^ were used in previous studies as biochar to remove HMs from aqueous solutions. Urban pruning waste (UPW) includes a large volume of pruned branches of trees and shrubs collected from urban areas and transported to waste disposal sites. Disposal and open-air burning are common practices for UPW management in many regions worldwide. These are serious environmental problems due to the emission of greenhouse gases, as well as a risk to public health^27^. UPW can be used to biochar production as a low-cost, high-efficacy adsorbent for the removal of contaminants such as HMs from aqueous solutions to eliminate environmental problems associated with the disposal and/or burning of this type of waste^28^.

Although the concentration of HMs decreases with the increasing aging of landfill sites, but they are high enough to be regarded as a problem with the continuous strengthening of the discharge standards in many countries. When the treatment of stabilized leachate is considered, physicochemical approaches are found to be more suitable in removing HMs. To the best of our knowledge, no study has focused on UPW-derived biochar to remove HMs in stabilized MSW landfill leachate, particularly the effects of pyrolysis temperature and biomass particle size on the adsorption efficiency of Pb, Cd, and Mn. Therefore, in this study physical and chemical features of UPW-derived biochar obtained at different pyrolysis temperatures and particle sizes were characterized, and the effects of pyrolysis temperature and particle size on the removal efficiencies of Pb, Cd, and Mn in old landfill leachate at different pH solution, contact times and adsorbent dosages were investigated statistically.

## Materials and methods

### Landfill site description and leachate sampling

The leachate samples used in this study were collected from a 20-year-old MSW landfill (Anjilsi landfill site, ALS) located at 36.3600° N, 52.5475° E in Mazandaran province, Iran. Every day ca. 250 tons of collected MSW are transferred to the ALS, which means over 91 thousand tons per year. MSW composition in ALS comprises 65.37% food waste, 8.13% paper and cardboard, 7.48% plastic, 1.87% ferrous metals, 2.32% non-ferrous metals, 1.25% glass, 1.39% wood, 1.14% polyethylene terephthalate (PET), 1.10% textile, 0.51% rubber, and 9.44% other wastes^29^. The presence of a high content of organic waste in the MSW composition leads to a daily production of 125 m^3^ landfill leachate in ALS. A 32 × 73 m pond with a depth of 8–10 m has been formed next to the ALS due to the improper waste disposal and the lack of leachate collection and treatment system^30^. The inorganic fraction of MSW includes various metallic materials such as discarded wires, fluorescent lamps, thermometers, batteries, and old computers and phones that are transferred to the ALS in the absence of proper technical and cultural infrastructure for source separation and recycling. This can increase the concentration of HMs in the ALS leachate.

Samples were collected from the leachate pond and immediately transferred to the laboratory. Leachate samples stored at 4 °C, and the associated tests were conducted on them until a maximum of three days after transferring to the laboratory to prevent any potential chemical and biological changes^31^.

### Leachate characterization

The characteristics of the leachate samples including pH, electrical conductivity (EC), 5-day biochemical oxygen demand (BOD_5_), chemical oxygen demand (COD), and HM concentration were determined based on the standard methods for the examination of water and wastewater^31^. pH and EC were measured using pH meter (Metrohm 691, Swiss) and EC meter (WTW inoLab Cond 7110, Germany), respectively. For BOD_5_ determination the container containing the sample was placed in an incubator (LIEBHERR FKU 1800, Austria) and the BOD_5_ was measured by a BOD meter (Aqualytic BD600, Germany). The COD was determined using the reactor digestion method by a spectrophotometer (HACH DR5000, USA). HMs were measured using an inductively coupled plasma mass (ICP-MS) spectrometer (Varian Vista- MPX, Australia).

### Feedstock and biochar preparation

Based on the current knowledge, no study has been reported in literature related to the use of wild privet (*Ligustrum vulgare*, LV) as biosorbent for the removal of HMs. So, LV biomass waste materials were selected to produce biochar in this study. LV is a species of Ligustrum native to central and southern Europe, north Africa and southwestern Asia, from Ireland and southwestern Sweden south to Morocco, and east to Poland and northwestern Iran. The LV residues were collected from Mazandaran province, Iran. The feedstock materials were rinsed with deionized water (DI) several times and dried at ambient temperature (25 ± 2 °C). The feedstocks were crushed to a grain size of 1–2 mm and 63–75 µm using a laboratory planetary ball mill (Amin Asia Fanavar Pars NARYA-MPM 2 * 250 H, Iran) and then oven dried at 105 °C for 24 h. Crushed dry samples were loaded into a muffle furnace (Sanat Ceram PC-21A, Iran) under oxygen-limited conditions. The heating rate was 10 °C min^−1^ with a residence time of 1 h at the targeted peak temperatures of 400, 550, or 700 °C. After the pyrolysis process was completed, the samples were allowed to cool down in the furnace overnight. After cooling, the biochar samples were stored in a desiccator for subsequent analyses. The resulting biochars were designated by their particles size and peak temperature based on Table 1.

**Table 1:** Coded level of produced biochars under different pyrolysis temperatures and particle sizes.

Feedstock	Particle size	Pyrolysis temperature (°C)		
		400	550	700
*Ligustrum vulgare* (LV)	1–2 mm	Lv400-1	Lv550-1	Lv700-1
	63–75 µm	Lv400-63	Lv550-63	Lv700-63

### Biochar characterization

The proximate analysis was conducted to determine moisture content (ASTM D2867-09), volatile matter, VM (ASTM D5832-98), and ash (ASTM D2866). The fixed carbon (FC) was calculated by the difference in moisture, VM, and ash contents. Biochar yields are given as feedstock recovery and expressed as a percentage of weight of dry feedstock. The pH and EC of the biochar samples were measured by combining biochar with DI water in a mass ratio of 1:10 using pH meter (Metrohm 691, Swiss) and EC meter (WTW inoLab Cond 7110, Germany), respectively. The elemental analysis (carbon, C; hydrogen, H; nitrogen, N; and oxygen-O) of the obtained samples was performed using an elemental analyzer (Thermo Finnigan Flash EA 1112 series, USA). Molar H/C and O/C ratios were calculated from the elemental analysis for supportive indications of aromaticity and polarity. The Brunauer–Emmett–Teller (BET) SSA was determined by N_2_ gas sorption analyzer (Micromeritics TriStar II Plus, USA). Prior to analysis, the samples were degassed for 18 h under vacuum at 300 °C. The functional groups were detected using the Fourier-transform infrared spectroscopy, FTIR (ABB Bomem MB-Series, Canada) in the scanning range of 4000–400 cm^−1^. Pellets made according to the procedure established by the standard test method, ASTM E 1252-98(21). Surface electronic states were analyzed via Thermo Scientific K-Alpha (Thermo Fisher, USA). The surface morphology of materials was imaged using the field emission scanning electron microscope, FE-SEM (TESCAN MIRA3 XMU, Czech Republic) equipped with energy dispersive X-ray spectroscopy (EDS) detectors.

### Batch adsorption experiments

Batch adsorption experiments were performed in a series of 250 ml Erlenmeyer flasks containing 100 ml MSW landfill leachate. The effect of pyrolysis temperature (400, 550, and 700 °C) and adsorbent particle size (1–2 mm and 63–75 µm) on the removal of HMs ions was studied with a biochar dosage of 0.1 g L^−1^ and contact time of 24 h. The effect of solution pH was investigated by mixing the Lv700-63 sample (0.1 g L^−1^) with an initial pH of 2.0, 3.0, 4.0, 5.0, 6.0, and 7.0. The pH of the solutions was adjusted using 1 M HCl or 1 M NaOH. Kinetic experiments were carried out by mixing the working solution with 0.1 g L^−1^ of Lv700-63 for specific time intervals (0, 30, 60, 90, 120, 150, 180, 210, 240, 270, and 300 min). Equilibrium experiments took place for varying Lv700-63 dosage (0, 0.1, 0.5, 1, 1.5, 2, 2.5, 3, 3.5, 4, 4.5, and 5 g l^−1^) for the contact time determined by the kinetic experiments. Note that the operating variables were use on the basis of the literature review and authors own preliminary studies. In this study, no adjusting pH was conducted and the pH of landfill leachate which used as an adsorbate solution was maintained as received at ALS.

The mixtures were shaken at 120 rpm using an orbital shaker (Fan Azma Gostar TM52E, Iran) at room temperature (25 ± 2 °C). Obtained mixtures were centrifuged (Hettich UNIVERSAL 320 R, Germany) for 15 min at 6000 rpm to separate liquid and solid phases. All samples were filtered through a Whatman 0.45 µm prior to analysis as to minimize the interference of carbon fines with the analysis. The HM concentrations in the filtrates were determined using ICP-MS (Varian Vista- MPX, Australia). The adsorption capacity at the equilibrium, q_e_ (mg g^−1^) and the adsorption removal efficiency, R (%) was calculated as:

1 $$ q_{e} = \frac{{\left( {C_{0} - C_{e} } \right) \times V}}{M} $$

2 $$ R(\% )\left( {\frac{{\left( {C_{0} - C_{e} } \right)}}{{C_{0} }} \times 100} \right) $$

where C_0_ (mg L^−1^) is the initial HM concentration, C_e_ (mg L^−1^) is the HM concentration at equilibrium, m (g) is the mass of used biochar and V (L) is the volume of the adsorption solution.

### Kinetic and isotherm adsorption models

The adsorption kinetics was modeled by using pseudo first-order^32^ and pseudo second-order^33^ kinetics. The linear form of the pseudo first-order model is expressed by Eq. (3).

3 $$ \ln (q_{e} - q_{t} ) = \ln q{}_{e} - k_{1} t $$

where q_e_ is the amount of HM ions adsorbed at equilibrium (mg g^−1^), q_t_ is the amount of HM ions adsorbed at time, t, and K_1_ is the pseudo first-order rate constant.

Linear form of pseudo second-order kinetic is presented as Eq. (4).

4 $$ \frac{t}{{q_{{\text{t}}} }} = \frac{1}{{k_{{2{\text{p}}}} q_{e}^{2} }} + \frac{1}{{q_{e} }} $$

where K_2p_ is the pseudo-second order rate constant (g mg^−1^ min^−1^) and K_2p_q_e_^2^ or h is the initial adsorption rate (mg g^−1^ min^−1^).

The adsorption mechanisms of HMs were determined on the basis of three isotherm models, the Langmuir^34^, Freundlich^35^, and Temkin^36^. The linear form of Langmuir adsorption model was applied in accordance to Eq. (5).

5 $$ \frac{1}{{q_{{\text{e}}} }} = \frac{1}{{bq_{{\text{m}}} }}\frac{1}{{C_{{\text{e}}} }} + \frac{1}{{q_{{\text{m}}} }} $$

where q_e_ is the observed adsorption capacity at equilibrium (mg g^−1^), C_e_ is the HM equilibrium concentration (mg L^−1^), q_m_ is the maximum adsorption capacity (mg g^−1^), and b is the equilibrium constant for apparent energy of adsorption (L mg^−1^).

Adsorption efficiency can be predicted by a dimensionless constant separation factor or equilibrium parameter R_L_. The values of R_L_ were calculated from the following Eq. (6):

6 $$ R_{L} = \frac{1}{{1 + bC_{0} }} $$

If R_L_ > 1, then the adsorption is unfavorable; if R_L_ = 1, then the adsorption is linear; if 0 < R_L_ < 1, then the adsorption is favorable; and if R_L_ = 0, it is irreversible.

The linear form of the Freundlich adsorption model is given by Eq. (7):

7 $$ \ln \,q_{e} = \ln \,K_{F} + \frac{1}{n}\ln \,C_{e} $$

where K_F_ is the Freundlich constant related to adsorption capacity of adsorbent (mg^(1–1/n)^ g^−1^ L^1/n^) and n is the Freundlich equilibrium coefficient. 1/n is a Freundlich intensity parameter, which indicates the magnitude of the adsorption driving force or surface heterogeneity. Values of 1/n < 1 indicate the favorable adsorption, and values of 1/n > 1 represent unfavorable adsorption.

In linear form, the Temkin isotherm is represented by Eq. (8).

8 $$ q_{e} = B_{{\text{T}}} \ln \,K_{{\text{T}}} + B_{{\text{T}}} \ln \,C_{e} $$

where B_T_ is the variation of adsorption energy (J mol^−1^) and K_T_ is the equilibrium constant corresponding to maximum binding energy (L g^−1^).

### Statistical analysis

All experiments were conducted in triplicate, and the results are expressed as mean ± standard deviation. Linear methods were performed using the Microsoft Excel to compute parameters of adsorption isotherms and kinetics. Linear determination coefficient (R^2^) was applied to measure the matching degree between experimental and predicted data. The effects of pyrolysis temperature and adsorbent particle size on the removal efficiency of HMs on biochar were statistically analyzed using the one-way analysis of variance (ANOVA) in SPSS statistics. Differences were considered significant at *p* < 0.05.

## Results and discussion

### Raw landfill leachate characteristics

The physicochemical properties of the raw MSW landfill leachate are summarized in Table 1. The high levels of BOD_5_ (3099 ± 214 mg L^−1^) and COD (29,673 ± 361 mg L^−1^) indicated the high organic load of the leachate. Moreover, the leachate age (more than 20 years), pH (7.84 ± 0.8), and BOD_5_/COD ratio (0.10 ± 0.005) designated that the landfill is in the methanogenic phase. Comparison of landfill leachate characteristics in the present study with leachate from MSW landfills in Spain^1^, Germany^5^, and USA^4^ showed that the ranges of COD, BOD_5_, and HM concentrations in the ALS are higher than those reported in the mentioned countries. Leachate characteristics are dependent on the waste composition, which is notably different between developed and developing countries^37,38^. Considering the wastewater discharge standard (Table 2), some metal concentrations (i.e., lead, Pb; cadmium, Cd; and manganese, Mn) are higher than the permissible limit. Therefore, they were chosen for the study.

**Table 2:** Characteristics of the raw leachate sample.

Parameter	Unit	Value	National discharge standard	
			Surface water	Agriculture and irrigation
pH	–	7.84 ± 0.8	6.00–8.50	6–8.50
EC	μS cm^−1^	33.41 ± 2.38	–	–
BOD_5_	mg L^−1^	3099 ± 214	50	200
COD	mg L^−1^	29,673 ± 361	100	200
BOD_5_/COD	–	0.10 ± 0.005	–	–
As	mg L^−1^	0.06 ± 0.02	0.1	0.1
Cd	mg L^−1^	1.81 ± 0.65	0.1	0.05
Co	mg L^−1^	0.03 ± 0.01	1	0.05
Cr	mg L^−1^	0.26 ± 0.10	0.5	1
Cu	mg L^−1^	0.05 ± 0.01	1	0.2
Mn	mg L^−1^	7.23 ± 0.09	1	1
Pb	mg L^−1^	4.94 ± 1.02	1	1

### Characteristics of biochar

#### Physicochemical properties and elemental composition

The effects of pyrolysis temperature and particle size on the physicochemical and elemental composition of biochar are presented in Table 3. The pyrolysis temperature significantly affects the quality of produced biochar. The yield, VM, H, O, and H/C and O/C ratios of the biochar decreased significantly with an increase in the pyrolysis temperature; whereas pH, ash, FC, and SSA significantly increased. However, pyrolysis temperature had no significant effect on the N content of the biochar (*p* > 0.05). Moreover, particle size had no significant effect on the biochar properties (*p* > 0.05), and the only difference was observed in the SSA. In this case, the SSA values of the biochars size 63–75 µm was significantly higher than the biochars size 1–2 mm.

**Table 3:** Basic properties, proximate analysis, and elemental composition of Lv biochars.

Properties	Biochar						
	Lv400-1	Lv550-1	Lv700-1	Lv400-63	Lv550-63	Lv700-63	
Basic properties	Yield (%)	29.44 ± 0.16	27.67 ± 0.29	26.35 ± 0.11	26.19 ± 0.08	23.51 ± 0.21	19.70 ± 0.15
	pH	9.35 ± 0.02	9.66 ± 0.08	9.93 ± 0.03	9.73 ± 0.05	10.14 ± 0.01	10.25 ± 0.01
	BET (m^2^ g^−1^)	2.15 ± 0.03	52.35 ± 1.27	229.44 ± 2.74	4.07 ± 0.08	94.28 ± 1.35	369.63 ± 1.93
Proximate analysis	VM (%)	30.15 ± 1.22	19.44 ± 0.53	6.36 ± 0.62	39.75 ± 0.95	27.61 ± 1.15	8.56 ± 0.76
	Ash (%)	0.94 ± 0.0	1.53 ± 0.3	1.86 ± 0.1	1.03 ± 0.4	1.75 ± 0.2	2.17 ± 0.5
	FC (%)	68.91 ± 0.73	79.03 ± 1.05	91.78 ± 0.58	59.22 ± 1.12	70.64 ± 0.97	89.27 ± 0.67
Elemental composition	C (%)	65.26 ± 0.26	74.01 ± 0.91	77.65 ± 0.14	69.75 ± 0.67	78.63 ± 0.34	85.11 ± 1.03
	H (%)	4.23 ± 0.23	3.07 ± 0.04	1.96 ± 0.01	3.68 ± 0.05	2.17 ± 0.16	1.65 ± 0.03
	N (%)	0.22 ± 0.01	0.37 ± 0.10	0.31 ± 0.06	0.18 ± 0.04	0.19 ± 0.01	0.16 ± 0.03
	O (%)	30.29 ± 0.63	22.55 ± 0.41	20.08 ± 0.34	26.39 ± 0.72	19.01 ± 0.65	13.08 ± 0.39
Atomic ratio	H/C	0.77 ± 0.26	0.49 ± 0.12	0.30 ± 0.15	0.63 ± 12	0.33 ± 0.23	0.23 ± 0.14
	O/C	0.34 ± 0.10	0.22 ± 0.13	0.19 ± 0.12	0.28 ± 0.13	0.18 ± 0.14	0.11 ± 0.09

Mean ± standard deviation; VM: volatile matter, and FC: fixed carbon.

The ash content of biochar increased with an increase in pyrolysis temperature (Table 3) because of the destruction of lignocellulosic materials and increasing the concentration of minerals. Increase in ash content with increasing pyrolysis temperature was also reported by Wei et al.^40^ who observed that the ash content increased from 5.38% in the biochar pyrolysis temperature of 300 °C to 12.23% in the biochar pyrolysis temperature of 700 °C^39^. As pyrolysis temperature increased, pyrolytic volatile materials were converted to low-molecular-weight organic compounds and different gases, which ultimately led to increase of the ash content and decrease the biochar yield (Table 3). In this regard, Egbosiuba^41^ showed that increasing the pyrolysis temperature from 300 to 600 °C decreased the biochar yield from 68.59 to 44.36% as a result of the less formation of the aliphatic compounds and less release of pyrolysis gases (e.g., methane, CH_4_; dihydrogen, H_2_; and carbon dioxide, CO_2_) at lower temperature^40^. Kim et al.^42^ demonstrated that the pH of biochar increases from 6.88 to 7.26 with the increase of temperature (400–700 °C)^41^ which are close to the finding of this study (Table 3). The alkalinity of biochar might be influenced by the release of alkali salts and alkali earth metals from the feedstock with increasing pyrolysis temperature^42^.

An increased in the FC content of biochar produced at higher pyrolysis temperatures (Table 3) can be associated with the thermal decomposition of feedstock and released of volatile substances^43^. The change in the H and O contents in the studied biochar was opposite to that of FC content which were decreased with increasing pyrolysis temperature due to dehydration and deoxygenation of hydroxyl functional groups and oxygen bonds during pyrolysis process and release low-molecular-weight compounds containing H and O^44^. Some previous reports^45,46^ demonstrated that there is no consistent change with biochar N content with pyrolysis temperature. The results corresponded to findings of this study (Table 3). The C content increase with an increase in pyrolysis temperature; so, the H/C and O/C atomic ratios in the resulting biochar decreased consistently^47^. The trend in the H/C and O/C atomic ratios changes is in agreement with the results of the present study (Table 3). A decrease in H/C and O/C atomic ratios indicated the dehydration process and decarboxylation reactions, respectively; and both of them demonstrated the development of the biochar's aromaticity and its associated reactions^18^.

Table 3 represents that the LV biochars made at high temperature (i.e., 700 °C) have larger SSA than the biochars derived from different industrial and agricultural wastes such as red mud 100.80 m^2^ g^−1^^26^, sewage sludge 99.57 m^2^ g^−1^^25^, corncob 53.71 m^2^ g^−1^^48^, and wheat straw 24.5 m^2^ g^−1^^21^ at same pyrolysis temperature. The SSA of the biochar increased with pyrolysis temperature (Table 3). It might be as a result of accelerating the evaporation process of organic compounds at temperatures above 400 °C which led to considerable increase in biochar porosity and SSA^49^. Chatterjee et al.^50^ reached similar results and observed that the increase in pyrolysis temperature from 500 to 700 °C led to the increase in SSA of biochar from 4.58 to 107 m^2^ g^−1^. Albalasmeh et al.^15^ stated that a decrease in the feedstock particle size increase the microporosity of the produced biochar^20^. Furthermore, some research studies reported that biochar produced from feedstock with smaller particle sizes has larger external surface areas^51,52^.

### FE-SEM and EDS analysis

**Figure 1:**
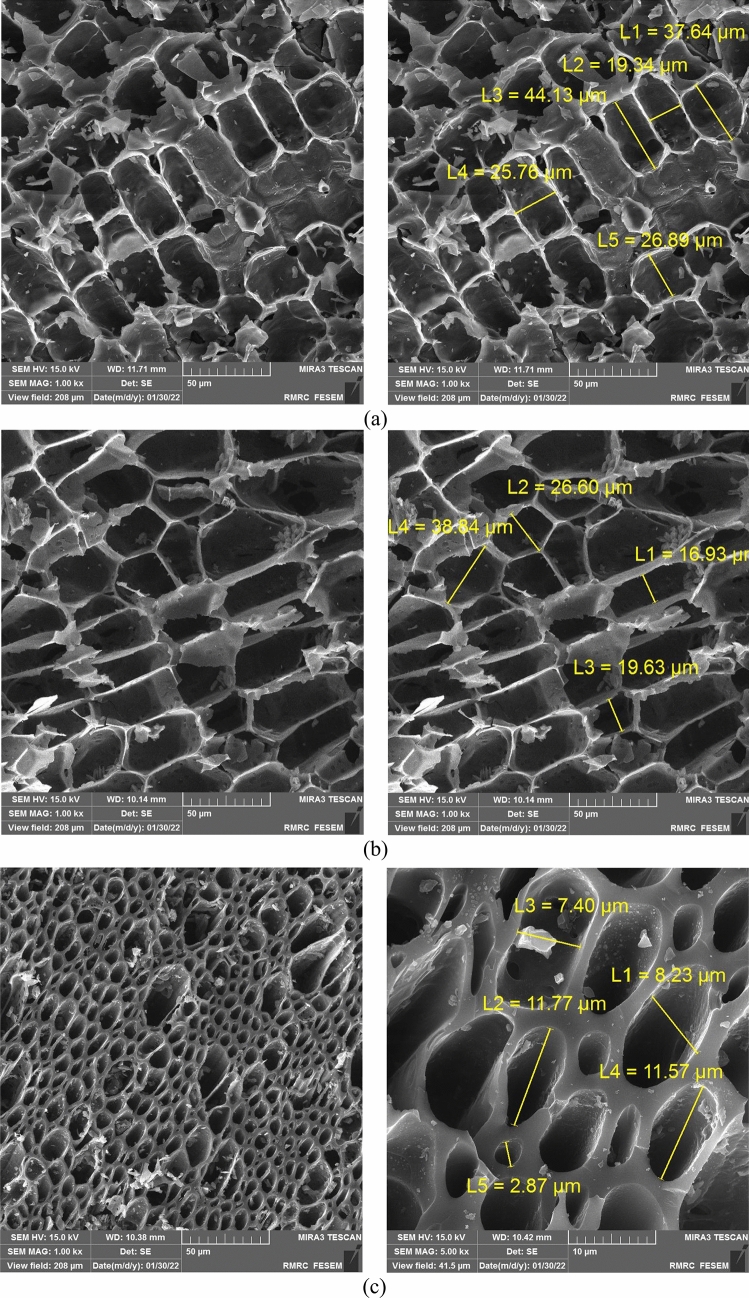
FE-SEM images of (**a**) Lv400-1, (**b**) Lv550-1, and (**c**) Lv700-1.

**Figure 2:**
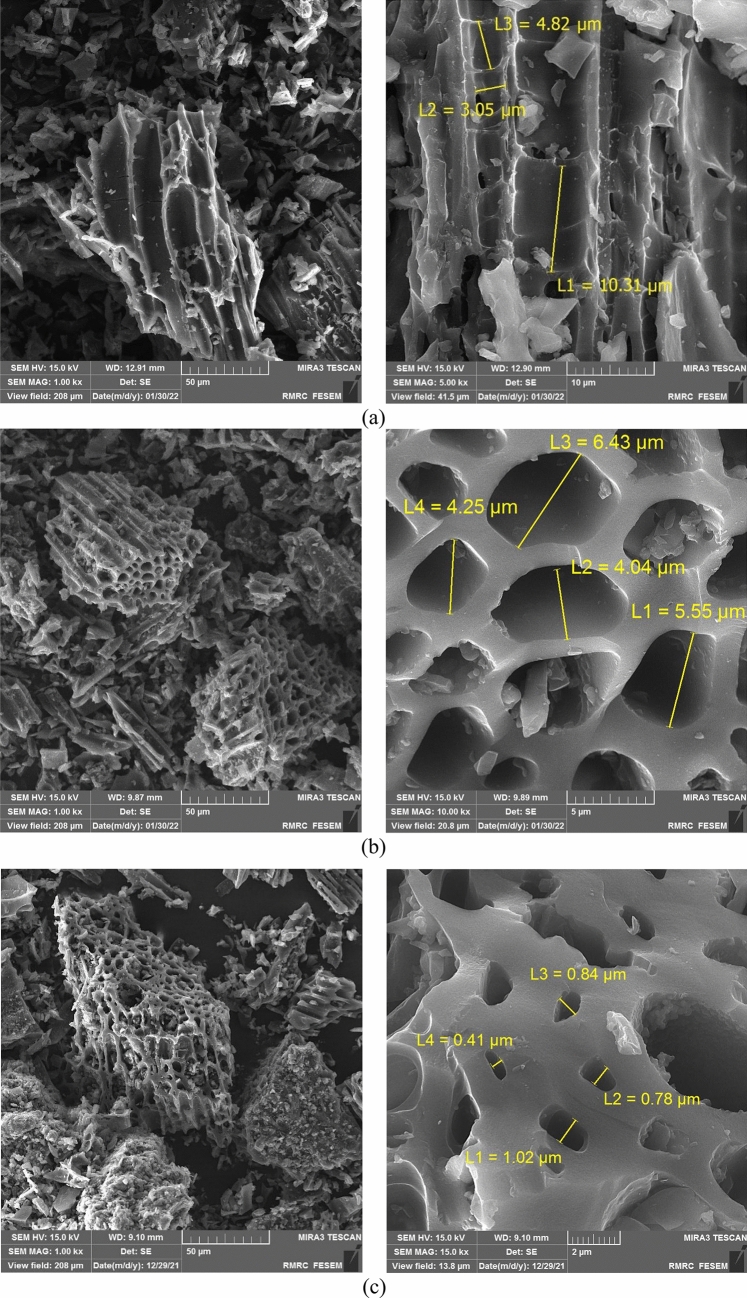
FE-SEM images of (**a**) Lv400-63, (**b**) Lv550-63, and (**c**) Lv700-63.

Figures 1, 2 show the FE-SEM images of biochar samples. Biochars prepared at a pyrolysis temperature of 400 and 550 °C demonstrated a compact surface containing very few pores, while produced biochars at 700 °C had a loose and porous structure (Figs. 1, 2). Increase in biochar porosity was mainly due to the speeding up evaporation and removing VMs^53^ which was also confirmed by the larger SSA for biochars produced at a high pyrolysis temperature (Table 3). The adsorbent with small particle size was characterized by fine porosity and a large internal surface area, while the powder form has larger diameter pores and smaller internal surface area^10^ which is in agreement with FE-SEM results (Figs. 1, 2). These findings showed that pyrolysis temperature and feedstock particle size affected the biochar structure and pore size distribution.

The EDS analysis of biochar samples is shown in Supplementary Figs. S1, S2. Calcium (Ca), magnesium (Mg), and sulfur (S) were found only in the biochars produced at the pyrolysis temperature of 400 °C, indicating that Ca, Mg and S were lost during biochar manufacture. The higher boiling point of Ca and Mg is the reason for the lower releasing of them during the pyrolysis process compared with an element such as K^45^. EDS analysis showed that C and O contents were increased and decreased, respectively with an increase in the pyrolysis temperature (Supplementary Figs. S1, S2). This was confirmed by the elemental analysis (Table 3). Changes in the nutrient concentration with increasing the pyrolysis temperature can be attribute to the partitioning and/or realizing the elements at high temperatures^49^. Moreover, increasing the pH with pyrolysis temperature (Table 3) may led to decrease the solubility of the elements. The particle size did not show a statistically significant main effect on the elemental composition of biochars (*p* > 0.05) (Supplementary Figs. S1–S2).

### FTIR analysis

**Figure 3:**
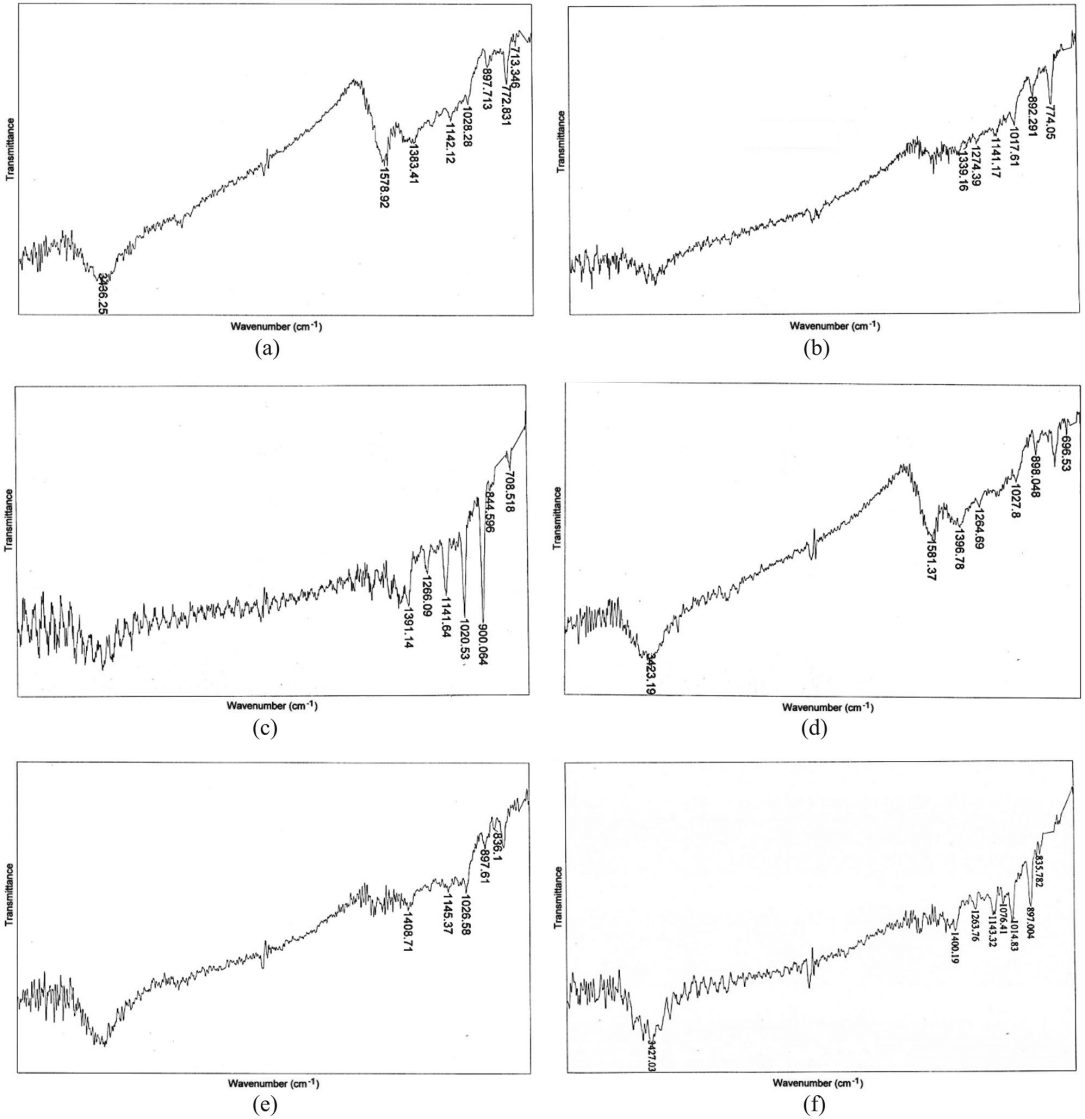
FTIR spectra of Lv400-1 (**a**), Lv550-1 (**b**), Lv6700-1 (**c**), Lv400-63 (**d**), Lv550-63 (**e**), and Lv700-63 (**f**) biochar samples.

The FTIR spectra of biochars is illustrated in Fig. 3. Biochar samples with a particle size of 1–2 mm present the various functional groups such as O–H stretching, C=C stretching, S=O stretching, C–H bending, O–H bending, C–N stretching, C–O stretching, C–F stretching, C=C bending, and C–Cl stretching. The prepared biochar samples with particle sizes of 63–75 µm show the peaks which are related to the presence of O–H stretching, C=C stretching, O–H bending, O–H bending, C–O stretching, S=O stretching, C–O stretching, C–O stretching, C–F stretching, C–H bending, C=C bending, and C=C bending functional groups.

Biochars produced at pyrolysis temperature of 400 °C presented the intense peaks of O–H, C=C, C–H, S=O, and C–O functional groups. The O–H and C=C peaks of biochars decreased in the intensity or disappeared with the increasing pyrolysis temperature from 400 to 700 °C. The functional groups were dehydrated upon increases in the pyrolysis temperature, whereby aliphatic bonds were converted to aromatic bonds which become stable graphene. High pyrolysis temperature promoted the deformation of hydrogen bonds in hydroxyl functional groups and hydrocarbons and progress of carbonization reaction^15^. Furthermore, thermal decomposition of cellulose and lignin of the biomass with increasing temperature led to decrease the polar functional groups and thus, increasing the aromatic carbon structures^51^. Although some minor variations in the type of functional groups present, the particle size seems to have very little effect.

### Adsorption of HMs from MSW landfill leachate

#### Effect of pyrolysis temperature and particle size

**Figure 4:**
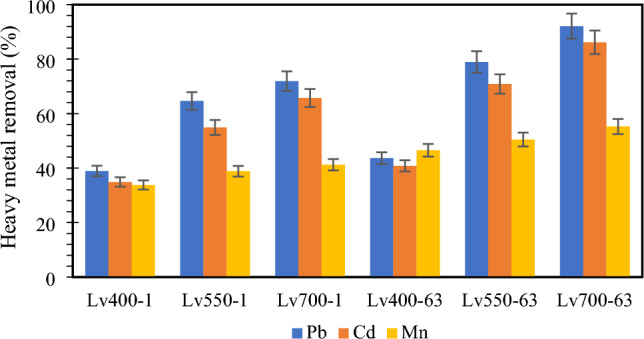
Effect of pyrolysis temperature and particle size on removal efficiency of HMs onto UPW biochar samples.

The removal efficiencies of HMs by UPW biochar samples at different pyrolysis temperatures and particle sizes are shown in Fig. 4. Operating conditions affected the removal efficiencies of HMs. The adsorption percentage of Pb, Cd, and Mn increased with increasing pyrolysis temperature from 400 to 700 °C and decreasing the adsorbent particle size from 1–2 mm to 63–75 µm. It showed the biochar pyrolyzed at 700 °C with particle size of 63–75 µm (i.e., Lv700-63) had the highest removal efficiency for Pb (92.07%), Cd (86.14%), and Mn (55.25%) from MSW landfill leachate (Fig. 4). This, the optimal adsorbent Lv700-63 was selected for HMs adsorption.

The adsorption efficiency of HMs onto biochar depends on physicochemical properties of the adsorbent which are affected by the production conditions. As pyrolysis temperature increased, the aromatic structure and polar functional groups were increased and decreased, respectively (Fig. 3). High pyrolysis temperature led to release more non-carbonaceous materials^39^ and affected the absorption efficiency performance (Fig. 4). Increasing pyrolysis temperature led to increase the C content and decrease the VM (Table 3) because of the decomposition of cellulose and hemicelluloses, which increased the biochar porosity (Figs. 1, 2) and HMs adsorption from MSW landfill leachate.

The adsorption efficiency of HMs increased with particle sizes decreasing from 1–2 mm to 63–75 µm. This was confirmed by the BET SSA of biochar samples (Table 3) which showed the smaller particle size increasing the available surface area for adsorption. As shown in Figs. 1, 2 the internal surface area increased with decreasing particle size, whereby the tendency of participate in chemical reactions tended to increase. Moreover, the electrical charge density per unit area of a surface increased with the decreasing particle size due to the constant electrical charge^54^, which gave high rates of adsorption (Fig. 4).

#### Effect of pH

**Figure 5:**
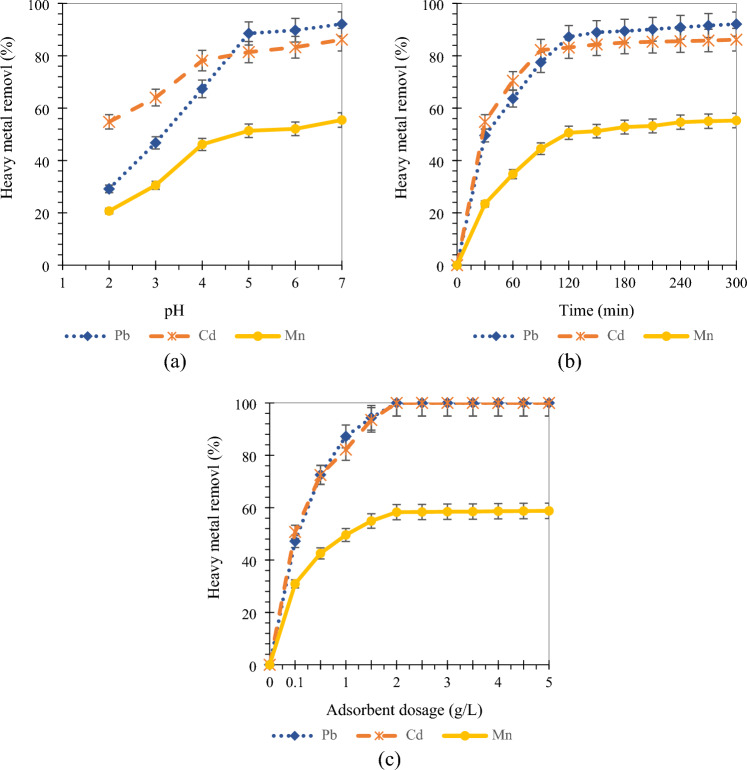
Effect of pH (**a**), contact time (**b**), and adsorbent dosage (**c**) on removal efficiency of HMs onto Lv700-63.

The effect of the pH of a solution is a major factor that determines the biosorption property of HM ions removal from an aqueous system. The pH of solution has a significant impact on metal uptake since it determines the surface charge of adsorbent, solubility of the HM ions and the degree of ionization and speciation of adsorbate^10^. Pb, Cd, and Mn adsorption increased with increasing solution pH from 2.0 to 7.0 (Fig. 5a). Thus, the optimal solution pH of 7.0 was selected for Pb, Cd, and Mn adsorption by Lv700-63. Similar results have been reported by previous studies. For example, Priya et al.^13^ reported that the maximum efficiency of Pb adsorption is obtained at 6.0 pH by the rice husk biochar. Also, Wand et al. (2019) revealed that the percentage removal of Cd by the Maize Straw increased from 37.3% to 96.7% as the initial pH increased from 2 to 6.5^19^. At a low pH, the H^+^ concentration is high, which can result in competition with HM ions for surface adsorption sites^22^. Therefore, high pH can increase Pb, Cd, and Mn adsorption efficiency.

#### Effect of contact time

The effect of contact time on HMs adsorption is presented in Fig. 5b. It is clear from Fig. 5b that the adsorption of HMs increased with prolonging the contact time. Pb and Mn equilibrium were achieved in 120 min onto Lv700-63. The removal efficiency of Pb and Mn at contact time of 120 min was 87.20 and 50.59%, respectively. Cd removal rate was faster compared to Pb and Mn and was achieved in 90 min. Maximum percentage removal Cd was found to be 82.16% using this variable of contact time. Kinetic equilibrium times determined contact times for the subsequent adsorption isotherm studies.

The results indicated that the Pb, Cd, and Mn adsorptive removal onto Lv700-63 increased with the contact time increasing (Fig. 5b). It is plausible to suggest that by increasing the contact time, there would be a more chance for surface interactions between the Lv700-63 biochar and metal ions^10^. The curves adsorption of Pb, Cd, and Mn (Fig. 5b) can be divided into three distinct regions; rapid adsorption during the early stage, gradual adsorption till the equilibrium state, and a plateau. Preliminary, metal adsorption sharply increased with enlarges with time. This is probably due to the availability of readily accessible sites of Lv700-63 are more at the beginning of the adsorption process^25^. Thereafter, active sites of Lv700-63 were saturated by metal ions and other existing contaminants in landfill leachate and the rate of adsorbate-adsorbent interaction becomes constant^14^.

#### Effect of adsorbent dosage

The effect of adsorbent dosage on percentage removal of HMs is illustrated in Fig. 5c. As shown in Fig. 5c, the percentage removals of Pb, Cd, and Mn were increased with increasing dosage of adsorbent up to 2 g L^−1^ and removals were constant thereafter, which revealed that equilibrium attainted at 2 g L^−1^ for metal ions. The removal efficiency of Pb and Cd by Lv700-63 was 100% for the adsorbent dosage of 2 g L^−1^. Maximum percentage removal of Mn was found to be 58.29% using this variable of adsorbent dose. So, the Lv700-63 dosage of 2 g L^−1^ was determined as the optimum value to Pb, Cd, and Mn removal from MSW landfill leachate.

At higher concentrations of adsorbent, the accessibility of surface-active sites increased, which increases adsorption percentage of HMs^55^. By increasing the Lv700-63 dosage more than 2 g L^−1^, the HMs adsorption efficiency was almost constant. This may be due to the increasing adsorbent dosage, decreased the saturation of active sites which causes the number of covered sites per unit mass of adsorbent to fall and led to a decrease in adsorption capacity^13^. Furthermore, increasing Lv700-63 dosage in MSW landfill leachate caused particle overlapping which reduced the SSA and increased diffusion path length^17^.

### Adsorption kinetics and isotherms

The fitting results of the pseudo first-order and pseudo second-order parameters are presented in Table 4. Correlation coefficients (R^2^) of the pseudo-second-order (R^2^ = 0.99) were higher than those of the pseudo first-order (0.73 ≥ R^2^ ≥ 0.98). The experimentally calculated q_e_ were well matched with the theoretical q_e_ values of the pseudo-second-order kinetic (Table 4). Therefore, it concluded that the pseudo second-order model describes the system well for all Pb, Cd, and Mn ions. This finding indicates that the rate-limited step is chemisorption involving exchange of the metal ions with functional groups in the adsorbent during valence forces^21,56^.

**Table 4:** Kinetic model parameters for the metal ions adsorption onto Lv700-63.

HM ions	q_e,exp_	Pseudo first-order			Pseudo second-order			
		q_e,cal_	k_1_	R^2^	q_e,cal_	k_2p_	h	R^2^
Pb	4.877	0.442	0.032	0.93	4.631	0.663	0.022	0.99
Cd	1.492	0.094	0.046	0.73	1.376	1.714	0.020	0.99
Mn	7.255	0.963	0.026	0.98	7.328	0.041	0.020	0.99

Langmuir, Freundlich, and Temkin isotherm parameters and their values are presented in Table 5. It is clear that Langmuir model (R^2^ = 0.99) was closely fits to experimental data than other investigated isotherm models i.e., Freundlich (0.82 ≤ R^2^ ≤ 0.93) and Temkin (0.64 ≤ R^2^ ≤ 0.82). Furthermore, the values of 0 ≤ R_L_ ≤ 1, indicating that the Langmuir adsorption is appropriate for adsorption process of Pb, Cd, and Mn on Lv700-63. Langmuir model assumes that there are a finite number of binding sites on the adsorbent which are homogeneously distributed over its surface^8^. Only a single monolayer is formed and there was no interaction between the adsorbed molecules^11^. So, it suggests the monolayer coverage of Pb, Cd, and Mn on the flat surface of the Lv700-63 having homogeneous distribution of active sites available for adsorption on the biochar surface.

**Table 5:** Isotherm models parameters for the adsorption of HMs onto Lv700-63.

HM ions	Langmuir				Freundlich			Temkin		
	q_m_	b	R_L_	R^2^	1/n	K_F_	R^2^	B_T_	K_T_	R^2^
Pb	13.855	0.760	0.210	0.99	0.049	0.760	0.89	0.825	3.541	0.72
Cd	5.598	0.982	0.360	0.99	0.067	0.695	0.82	0.360	7.515	0.64
Mn	28.141	0.108	0.394	0.99	0.151	1.60 × 10^–5^	0.93	6.511	0.172	0.82

From the Langmuir isotherm model, the maximum adsorption capacity (q_m_) values for Pb, Cd, and Mn were 13.855, 5.598, and 28.141 mg g^−1^, respectively (Table 5). The Langmuir q_m_ of Lv700-63 is compared with that of other adsorbents for HMs removal from real and synthetic wastewaters in Table 6. As can be seen in Table 6, the adsorption capacity of Lv700-63 was higher than that of bagasse, potato peel, *Rhizopus oryzae*, coconut shell, bamboo, and *Malpighia emarginata* D.C. seed fibers for Pb, Cd, and Mn adsorption from landfill leachate and other industrial wastewaters. The high q_m_ values prove the effectiveness of Lv700-63 biochar in treating wastewater. However, it is noted that the adsorption capacity of the HMs in the synthetic aqueous solutions is higher than that in the real wastewaters; the reason behind this may be the treatment of effluent (with lesser concentration of HMs (0.035–12.77 mg L^−1^) than that of known solutions of metal ions containing higher concentrations of them (50–3000 mg L^−1^) (Table 6). Moreover, in real multicomponent wastewaters, metal ions interact with each other in synergistic, antagonistic or non-interactive manner and the adsorption capacity of adsorbent for HMs decreased as compared to aqueous single metal solutions^16,57^.

**Table 6:** Comparison of maximum adsorption capacity (q_m_) of some adsorbents for HM ions removal from real and synthetic wastewaters.

Biochar	HM ions	Initial concentration (mg L^−1^)	q_m_ (mg g^−1^)	Application	References
Bagasse	Pb	2.393	12.74	Battery manufacturing wastewater	^58^
Potato peel	Pb	2.520	0.0981	Metal plating industry	^59^
	Cd	0.149	0.0002		
*Rhizopus oryzae*	Pb	7.53	12.391	Shipyard wastewater	^2^
Coconut shell	Mn	2.66	0.0626	Landfill leachate	^60^
Bamboo (*Guadua amplexifolia*)	Pb	5	1.073	Landfill leachate	^61^
*Malpighia emarginata* D.C. seed fibers	Cd	0.035	0.124	Landfill leachate	^62^
	Pb	0.120	0.089		
*Ligustrum vulgare*	Pb	4.94	13.855	Landfill leachate	This study
	Cd	1.81	5.598		
	Mn	7.23	28.141		
Cherry kernel	Pb	50	69.75	Synthetic wastewater	^63^
Maize Straw	Cd	170	35.46	Synthetic wastewater	^19^
Peanut shell	Pb	200	57.19	Synthetic wastewater	^22^
	Cd	200	38.448		
Pomelo peel	Mn	3000	163.194	Synthetic wastewater	^64^
Sunflower (stems and baskets)	Mn	500	45.4	Synthetic wastewater	^65^

### Adsorption mechanisms

**Figure 6:**
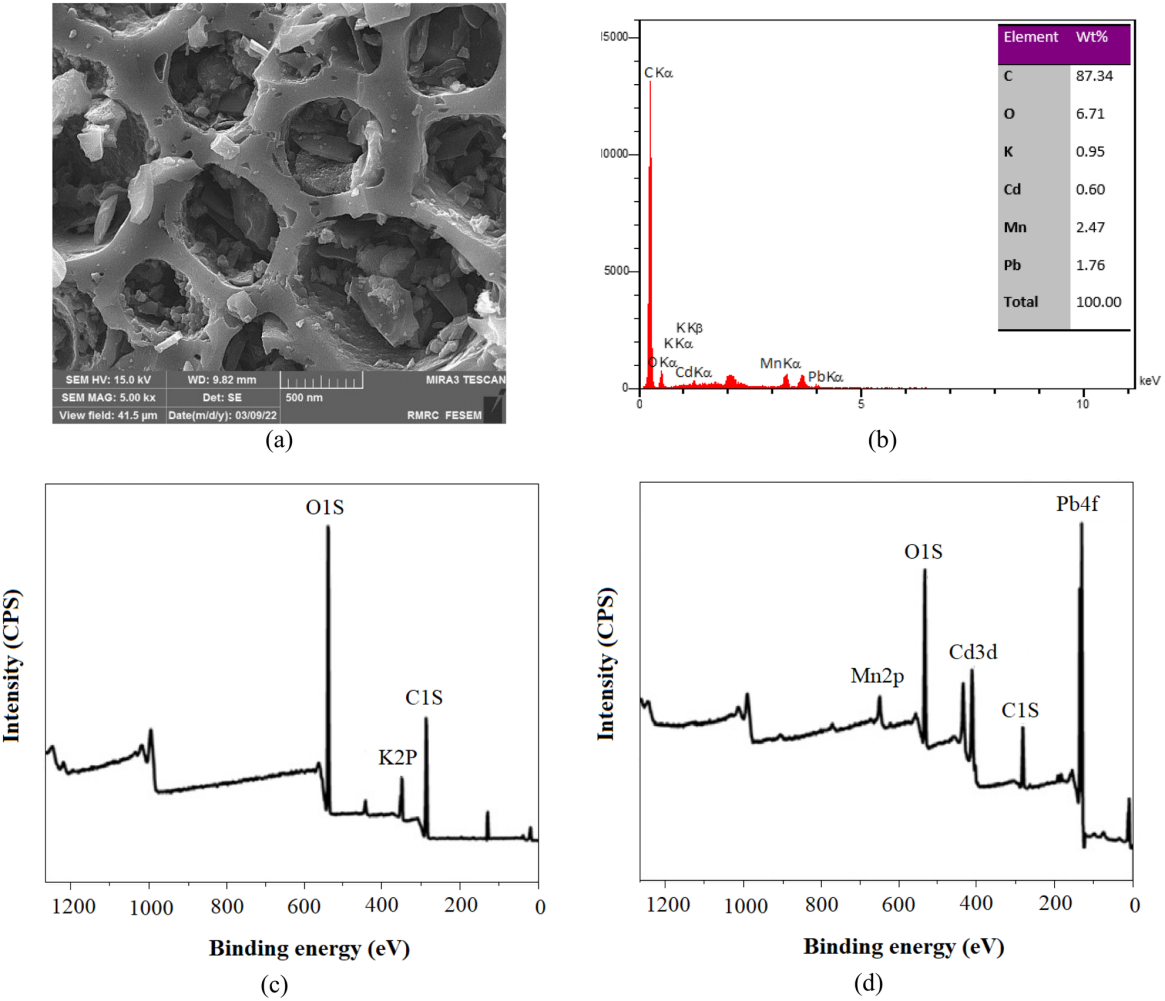
FE-SEM image (**a**) and EDS analysis (**b**) after adsorption and XPS spectra before (**c**) and after (**d**) adsorption of the Lv700-63.

Figure 2c showed a structure of heterogeneous and wide porous surface of Lv700-63, which are favorable properties when using the substance in the adsorption of HMs. Generally, rougher surface area and broadly distributed pores can offer an effective surface area and more opportunities for the binding of HM ions^55^. FE-SEM image of a Lv700-63 biochar sample after adsorption is illustrated in Fig. 6a. This image is representative of the pore filling occurrence due to the existence of mesopores and micropores in Lv700-63.

The FTIR results showed that Lv700-63 surface had oxygen-containing functional group of hydroxyls (Fig. 3). With the pyrolysis temperature increasing, the aromatic C-H and aromatic C=C peaks became more intense, indicating that more pronounced aromatic structure was formed in Lv700-63 biochar. The aromatic structure can provide π electrons, which have been reported to have the potential to bond HM cations strongly^12^. To determine the main functional groups of Lv700-63 that are responsible for HMs adsorption, FTIR spectra was recorded after adsorption (Table 7). The disappearance of the peaks corresponding to C=C and C–O form indicated surface complexation of HMs through delocalized π electrons^22^. The disappearance of the peaks at 1263.76, 1076.41 cm^−1^ (attributed to C–O stretching), and 835.78 cm^−1^ (attributed to 835.78 C=C bending) might be responsible for Pb, Cd, and Mn adsorption on Lv700-63 through Cπ–cation interactions.

**Table 7:** FTIR spectra of Lv700-63 after adsorption of Pb, Cd, and Mn.

IR peaks	Before adsorption	After adsorption	Assignments
1	3427.03	3314.51	O–H stretching
2	1400.19	1353.26	O–H bending
3	1263.76	Disappear	C–O stretching
4	1143.32	1146.54	S=O stretching
5	1076.41	Disappear	C–O stretching
6	1014.83	1015.57	C–F stretching
7	897.00	883.42	C–H bending
8	835.78	Disappear	C=C bending

There are usually many exchangeable metal ions (e.g., Ca^2+^, Mg^2+^, K^+^, and sodium, Na^+^) retained on the biochar, which could be exchanged by HMs in solution during the adsorption process^56^. The main elements of Lv700-63 biochar before adsorption comprise C, O, and K (Supplementary Fig. S2). The Lv700-63 might possess K_2_CO_3_ on the surface. The remarkable peak of Pb, Cd, and Mn appeared for the Lv700-63 after adsorption (Fig. 6b). After adsorption, HMs react with carbonate to form surface precipitation^66^. The EDS results show that after adsorption, K remains on the Lv700-63 surface. This means that HM ions were adsorbed on the Lv700-63 surface through the surface precipitation of (HM,K_2_)CO_3_^67^. This was confirmed by the FE-SEM image (Fig. 6a) that shows the phenomenon of surface precipitation onto Lv700-63.

Figure 6c,d shows the changes in chemical values of surface elements of Lv700-63 before and after adsorption. Spectral scans of Lv700-63 before and after adsorption demonstrate the presence of several distinct peaks of C1s (281.54 eV), O1s (543.27 eV), and K2p (380.72 eV) in Lv700-63 prior to adsorption, proving that Lv700-63 contains C, O, and K^68^. This is according to the EDS findings of the material (Supplementary Fig. S2). After adsorption (Fig. 6d) of HMs, XPS diffraction peaks appeared at 410.5, 134.7 and 643.18 eV, which belong to the XPS characteristic peaks of the orbital splitting peaks of Cd3d 3/2, Pb4f. 7/2, and Mn2p1/2 respectively^69^. It proved that Pb, Cd, and Mn were captured by Lv700-63. The EDS results are proven by facts that the peak intensity of K2p decreased after HMs were adsorbed, suggesting that Pb, Cd, and Mn may have exchanged ions during the reaction^8^.

## Conclusions

The results obtained in this study have revealed that biochar properties are essentially determined by pyrolysis temperature. The yield, VM, H, and O contents decreased with increasing pyrolysis temperature from 400 to 700 °C; while pH, EC, SSA, ash, FC, and O contents increased. Smaller particle sizes of feedstock increased SSA and had no significant effect on other biochar properties (*p* > 0.05). The FE-SEM images showed that the high temperature and smaller particle sizes led to develop porous structure at the surface of the biochar. Moreover, an increase in pyrolysis temperature from 400 to 700 °C led to a decrease in biochar surface functional groups.

The experimental results manifested that the adsorptive removal of Pb, Cd, and Mn from MSW landfill leachate onto Lv700-63 biochar increased with increasing the pyrolysis temperature, solution pH, contact time, and adsorbent dosage; while, adsorption rate of HMs increased with decreasing particle size. The adsorption of Pb, Cd, and Mn onto LV700-63 was favorably described by the Langmuir isotherm and pseudo second-order kinetic models suggesting a monolayer and chemisorption process. Adsorption mechanisms of Pb, Cd, and Mn on Lv700-63 biochar might involve surface complexation, π-electron coordination, and cation exchange. This study suggests that UPW-derived biochar might be used as an effective, low-cost and environmentally friendly adsorbent for Pb, Cd, and Mn removal in MSW landfill leachate.

### Supplementary Information

Supplementary Figures.

## Data Availability

The datasets generated and/or analyzed during the current study are available in the published article. The additional datasets available from the corresponding author on reasonable request.
