# Supplementary material for: Pb(ΙΙ), Cd(ΙΙ), and Mn(ΙΙ) adsorption onto pruning-derived biochar: physicochemical characterization, modeling and application in real landfill leachate
Source: Sci Rep. 2024 Feb 10;14:3426. doi: 10.1038/s41598-024-54028-6 (PMC11306770; doi:10.1038/s41598-024-54028-6)
Supplement: Supplementary file 1 — Supplementary Figures. [file 41598_2024_54028_MOESM1_ESM.docx]

**Pb(ΙΙ), Cd(ΙΙ), and Mn(ΙΙ) Adsorption onto Pruning-derived Biochar: Physicochemical Characterization, Modeling and Application in Real Landfill Leachate**

Maryam Rabiee Abyaneh^1,*^,Gholamreza Nabi Bidhendi^2^, Ali Daryabeigi Zand^2^

^1^Department of Environmental Engineering, Kish International Campus, University of Tehran, Kish, Iran

^2^Faculty of Environment, University of Tehran, Tehran, Iran

^*^Corresponding author:

E-mail address: rabiee.abyaneh.ma@ut.ac.ir

| 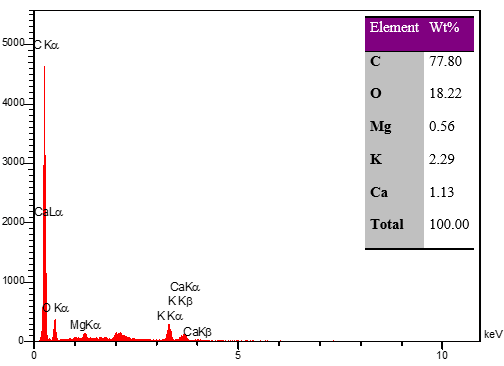 |
| --- |
| (a) |
| 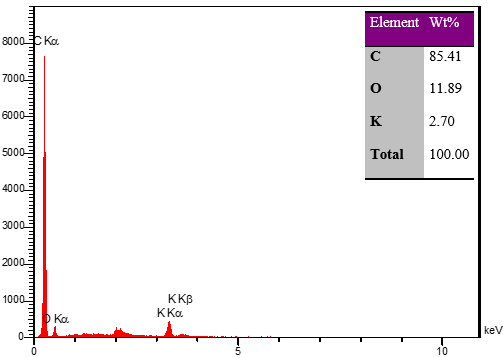 |
| (b) |
| 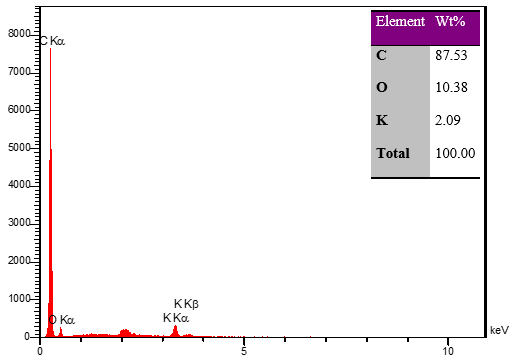 |
| (c) |

**Supplementary Figure S1.** EDS analysis of (a) Lv400-1, (b) Lv550-1, and (c) Lv700-1.

| 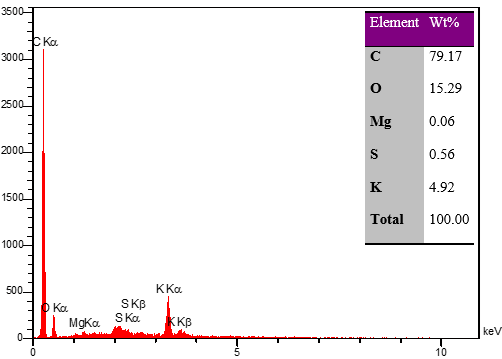 |
| --- |
| (a) |
| 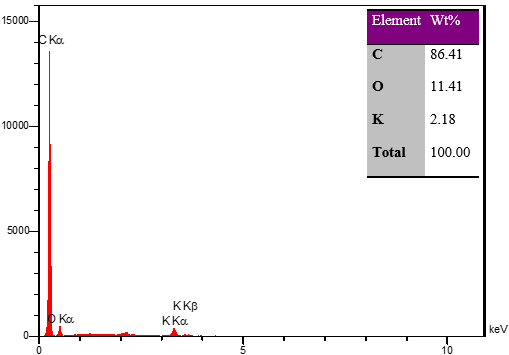 |
| (b) |
| 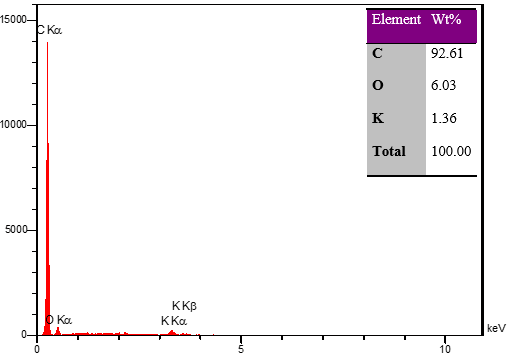 |
| (c) |

**Supplementary Figure S2.** EDS analysis of (a) Lv400-63, (b) Lv550-63, and (c) Lv700-63.
